# The gut virome: the ‘missing link’ between gut bacteria and host immunity?

**DOI:** 10.1177/1756284819836620

**Published:** 2019-03-25

**Authors:** Indrani Mukhopadhya, Jonathan P. Segal, Simon R. Carding, Ailsa L. Hart, Georgina L. Hold

**Affiliations:** Gastrointestinal Research Group, Division of Applied Medicine, University of Aberdeen, Foresterhill, Aberdeen, UK Gut Health Group, The Rowett Institute, University of Aberdeen, Foresterhill, Aberdeen, UK; St. Mark’s Hospital, Watford Road, Harrow, UK Imperial College London, South Kensington Campus, Department of Surgery and Cancer, London, UK; Gut Microbes and Health Research Programme, The Quadram Institute, Norwich Research Park, Norwich, Norfolk, UK Norwich Medical School, University of East Anglia, Norwich Research Park, Norwich, Norfolk, UK; St. Mark’s Hospital, Watford Road, Harrow, UK Imperial College London, South Kensington Campus, Department of Surgery and Cancer, London, UK; Microbiome Research Centre, St George & Sutherland Clinical School, University of New South Wales, Sydney, NSW 2217, Australia

**Keywords:** gut virome, gut microbiota, inflammatory bowel disease, host:microbiota interactions, microbial therapeutics, microbial dynamics

## Abstract

The human gut virome includes a diverse collection of viruses that infect our own cells as well as other commensal organisms, directly impacting on our well-being. Despite its predominance, the virome remains one of the least understood components of the gut microbiota, with appropriate analysis toolkits still in development. Based on its interconnectivity with all living cells, it is clear that the virome cannot be studied in isolation. Here we review the current understanding of the human gut virome, specifically in relation to other constituents of the microbiome, its evolution and life-long association with its host, and our current understanding in the context of inflammatory bowel disease and associated therapies. We propose that the gut virome and the gut bacterial microbiome share similar trajectories and interact in both health and disease and that future microbiota studies should in parallel characterize the gut virome to uncover its role in health and disease.

Key points

- The human intestinal microbiome represents one of the most complex ecosystems on Earth and has taken millions of years to coevolve.
- DNA and RNA viruses that collectively make up the intestinal virome outnumber bacterial cells by as much as 10:1, and include eukaryotic viruses which infect eukaryotic cells, endogenous retroviruses, bacterial viruses (i.e. bacteriophages) and archaeal viruses that infect archaea.
- Diet is an important and constant environmental and lifestyle factor that can influence the gut microbiome, including its viral component.
- Transkingdom interactions between virome components and bacteria highlights that there are additional layers of complexity to consider in terms of host–microbial homeostasis.
- Current microbial therapeutics including faecal microbial transplantation need to consider the contribution of the virome.
- Further research into the interconnectivity of the virome with other elements of the microbiome is essential to fully define the role of the gut virome in human health.

## Introduction

The human intestinal microbiota comprising bacteria, viruses, fungi, multicellular parasites and archaea represents one of the most complex ecosystems on Earth. It has coevolved over millions of years to help shape and influence human development, and in particular immune defences.^1^ The DNA and RNA viruses that collectively make up the intestinal virome are at least equivalent in number to bacterial cells,^2^ although on gut mucosal surfaces and within the mucus layers they may outnumber bacterial cells by 20:1.^3^ Each gram of human gut content is estimated to contain at least 10^8^–10^9^ virus-like particles (VLPs), the vast majority of which are crAssphage (cross-assembly phage), which are DNA phages belonging to the family Podoviridae (Box 1).^4,5^ Viruses have cross-kingdom interactions with other organisms within the microbiota that together with host genetic variation can change the host (human) phenotype. These virus-driven phenotypic changes can be beneficial to the host or increase the risk of disease.^6,7^ Currently, it is estimated that less than 1% of the virome has been sequenced, leaving the bulk of the virome yet to be characterized.^8^

**Box 1.** Getting access to the virome.

To study the virome, VLPs are separated from cellular components, usually using a combination of filtration, density centrifugation and enzymatic treatments to eliminate free nucleic acids.^9^ The nucleic acids are then sequenced and analysed. Compared to the bacterial component of the intestinal microbiome, the intestinal virome has been under-investigated and largely ignored, in large part due to the limited tools available for their identification and classification. Recent advances in high-throughput, next-generation sequencing has allowed detailed and in-depth analysis of microbial communities (metagenomics), leading to the identification of new microbial species, which has only recently been applied to the characterization of the virome.^10^ There are, however, too few reference viromes, and those that do exist are dominated by unknown sequences with 60–90% of the reads often lacking functional or taxonomic annotations (the ‘viral dark matter’).^2,11^ Currently, it is estimated that roughly 1% of the virome has been sequenced, leaving the bulk yet to be characterized.^8^

## Human gut virome: main players

### Eukaryotic viruses

Sequencing of eukaryotic viral communities in faecal samples from children has identified Picobirnaviridae, Adenoviridae, Anelloviridae and Astroviridae family members, and species such as bocaviruses, enteroviruses, rotaviruses and sapoviruses.^12^ In addition, disease-associated viruses such as herpesviruses, polyomaviruses, anelloviruses, adenoviruses, papillomaviruses, polyomaviruses, hepatitis B virus, hepatitis C virus and human immunodeficiency virus (HIV) are also present in the intestinal viromes of some individuals, indicating that the gastrointestinal (GI) tract contains viruses capable of infecting host cells. As the majority of humans remain asymptomatic it has been proposed that these pathogenic viruses (pathobionts) have become part of the metagenome of normal individuals, with the majority rarely causing disease and remaining dormant within the host.^6,13^ Experiments in germ-free and antibiotic-treated mice have shown that the bacterial microbiome can promote the replication and in some cases persistence of enteric viruses^13^ with the efficient transmission of mouse mammary tumour virus requiring intestinal bacteria.^14^ Interactions between viruses and bacteria, and other constituents of the intestinal microbiome, are therefore important in influencing the course and outcome of virus infections.^15^

### Bacteriophages

Microbial viruses modulate their bacterial hosts directly through affecting their mortality and through horizontal gene transfer, and indirectly by reprogramming host metabolism. The human GI tract contains an estimated 10^15^ bacteriophages (phages; the phageome) which may represent the richest concentration of biological entities on Earth.^16^ Phages can be functionally categorized based on the lifecycle they adopt after infecting host cells.^17^ Lytic (virulent) phages lyse the cells they infect by hijacking the host cell’s replication mechanism to package and produce more phages and lytic enzymes that cause cell lysis and the release of newly formed phages from the cell. By contrast, temperate phages incorporate their genetic material into the host cell chromosome as prophages and replicate alongside the host cell. In some instances, temperate phages do not integrate and exist as circular or linear plasmids within the host bacterial cell.^18^ A third category are pseudolysogenic phages, which upon infecting a cell neither integrate stably into the host genome nor co-opt cell replication machinery, or kill the host cell.^17^ Recently, analysis of the viral fraction of existing metagenomic studies identified a DNA phage called crAssphage which, due to limitations in our ability to identify phage sequences, had been overlooked in previous studies.^5^ CrAssphages are highly abundant in the gut microbiome, it has been predicted, based on host co-occurrence profiling that crAssphage infect *Bacteroides* species. However, our understanding of crAssphage is limited as they have only recently been isolated in culture.^5^

## Acquisition of gut virome communities over time

Bacterial microbiome communities are established at birth and evolve over time to become ‘adult-like’ bacterial communities.^19–22^ While our knowledge of gut bacterial communities is relatively informed, we have a poor understanding of gut virome acquisition. The absence of microscopically detectable VLPs in the first faecal samples (meconium) of newborns is consistent with the intestinal virome being predominantly acquired postnatally.^23^ Within a week of birth, VLP numbers reach 10^8^/g in faeces, with the initial colonizers originating from a combination of dietary, maternal and/or environmental sources.^23^ The infant virome develops in parallel with the bacterial microbiome, with evidence of contractions and shifts in the phage community with age as bacterial communities expand and diversify.^24^ By adulthood, an individuals’ virome reaches a peak, with 80% of viruses persisting over a 2.5-year period (Table 1).^12,24^ Eukaryotic viruses, including adenovirus, cytomegalovirus (CMV), herpes simplex virus, enterovirus, Epstein–Barr virus, respiratory syncytial virus and human parvovirus B19, have all been detected in amniotic fluid of healthy mothers delivering healthy babies.^25^ Transmission of viruses such as HIV, hepatitis, influenza, rubella, CMV and herpes zoster virus through the placenta or vaginally are also well recognized;^26^ however, the influence these viruses may have on the gut virome and the wider gut microbiome is unclear. Other factors known to affect the establishment of the infant gut microbiome include mode of delivery,^20,27^ breast *versus* bottle feeding^28^ and smoking.^29^ These factors have yet to be investigated and appreciated in terms of the gut virome.

**Table 1.** Virus communities within the human gut.

**Gut bacteriophages**	
Mostly double-stranded and single-stranded DNA phages:	
Myoviridae, Podoviridae, Siphoviridae, Inoviridae and Microviridae	
**DNA viruses:**	
*Double-stranded*	*Single-stranded*
Adenoviridae	Anelloviridae
Herpesviridae	Circoviridae
Iridoviridae	
Marseilleviridae	
Mimiviridae	
Papillomaviridae	
Polyomaviridae	
Poxviridae	
**RNA viruses**	
*Double-stranded*	*Single-stranded*
Picobimaviridae	Caliciviridae
Reoviridae	Astroviridae
	Virgaviridae
	Picornaviridae
	Retroviridae
	Togaviridae
**Definitive pathogenic eukaryotic viruses infecting the gut**	
Rotavirus, norovirus, astrovirus, adenovirus (serotypes 40 and 41), enterovirus (only adenovirus is DNA virus, rest are all RNA viruses).	

Lim and colleagues characterized changes to the gut virome in the first few months of life and found that the gut bacteriophage community structure was composed primarily of a rich and diverse collection of phages, with the majority deriving from the Caudovirales order.^26^ They also showed that eukaryotic viral population richness was low in infancy and expanded thereafter, while bacteriophage richness was greatest in early life and decreased with age;^24^ by 24 months of age there was a marked shift towards an increased relative abundance of Microviridae. In the infant, members of the Picornaviridae, Adenoviridae, Astroviridae, Anelloviridae, Reoviridae and Caliciviridae families were prominent but did not persist throughout early development.^24,30^ The increase in Microviridae species was not driven by a particular bacteriophage, with bacteriophage and bacterial richness being inversely correlated in an age-dependent manner.^24^ Overall, these findings suggest that the microbiome shifts from a high bacteriophage–low bacterial diversity community at 0 months towards a low bacteriophage–high bacterial diversity community by 24 months of age.^24^ It has been established that the gut microbiota is dominated by fewer bacterial species at this age, suggesting that bacteriophage diversity is unsustainable longitudinally because of this lower bacterial richness.

Shifts in the virome richness parallels the age at which the infant bacterial microbiome approaches its peak and an adult composition.^19,21^ which is consistent with phages playing a role in modulating bacterial community structure and function through their ability to lyse and kill host bacteria.^31,32^ This further supports the hypothesis that the gut bacteriome and virome follow similar developmental trajectories.

Differences in the gut virome between monozygotic twins sharing the same *in-utero* environment were explored by Reyes and colleagues,^33^ who identified virome differences between healthy twin pairs and twin pairs discordant for developing malnutrition. Specifically, they found that members of the Anelloviridae and Circoviridae could discriminate discordant from concordant healthy pairs,^33^ suggesting that specific virome signatures are associated with, although not necessarily mediators of, malnutrition. Unlike infant twins, adult twins harboured gut viromes substantially different from those of their co-twins or mother, consistent with environmental factors driving virome community structure and, by implication, function. Diet is an important and constant environmental and lifestyle factor^34^ that can influence the gut microbiome, including its viral component. The viromes of unrelated individuals consuming the same diet show gradual convergence and similar viral community structure.^35^ Thus, as with the bacterial component of the intestinal microbiome, the genetic constitution of an individuals’ virome is a reflection of their genome, lifestyle and behaviour, with medication and age being particularly important.^36^

Understanding the link between virome and other microbiome constituents is currently constrained by a lack of standardized protocols for virome analysis, which are required for efficient isolation and analysis. New developments in metagenomics,^37^ enrichment cultures^38^ and bioformatics^39,40^ tools are urgently required to improve our ability to define and characterize viromes.

## Environmental factors impact on the gut virome

Gut bacterial density changes over time under the influence of inherited and environmental factors.^19,21,41^ While gut bacterial densities change in response to environmental factors, the gut virome is relatively more stable within an individual.^33,42^ This has been demonstrated in a longitudinal assessment of an individual in whom more than 80% of the viral contigs remained stable over a period of 2.5 years.^12^ The effect of diet on the gut virome was investigated by Reyes and colleagues, who found that when individuals were put on similar diets, a large amount of variance existed between them in terms of their gut virome.^33^ Minot and colleagues challenged this theory and suggested that diet had an effect on the gut virome.^10^ They found that when patients were put on an identical diet, the gut virome composition became more similar, but not identical, between patients. Kim and colleagues found that mucosal and luminal viromes of obese mice fed a high-fat, high-sucrose ‘Western’ diet were significantly enriched with temperate phages of the Caudovirales order.^43^ The discrepancy in findings may represent the small study numbers, the constraints and variability in methodologies and subject material while exposing the relative lack of our understanding of the gut virome.

Other environmental exposures, such as antibiotic use and location, can substantially affect the gut virome.^44^ Antibiotics enrich phage-encoded genes that confer resistance via disparate mechanisms to the administered antibiotic, as well as encode genes that confer resistance to other unrelated antibiotics.^45^ However, another study has suggested that the data had inflated false positives due to relaxed thresholds for *in silico* detection of antibiotic resistance genes.^46^

## Interconnectivity of the virome with other elements of the microbiome and its role in human health

Transkingdom microbiome interactions between viruses and bacteria can influence host health and disease.^26^ Intestinal antiviral immunity is reliant on Gram-negative commensal-dependent NF-ĸB signalling, while enteric viral infection protects against intestinal damage and pathogenic bacteria.^8,13^ Transkingdom interplay adds therefore an additional layer of complexity in terms of host–microbial homeostasis.

By acting as a vehicle for horizontal gene transfer, phages can influence bacterial evolution, diversity and metabolism.^12,16^ Surprisingly phage populations appear more stable in faecal microbial transplantation (FMT) experiments compared to bacterial communities.^47^ The pathways and mechanisms of transfer of phage-encoded genes including antibiotic resistance^48^ or virulence factors^49^ requires a more complete understanding of the functionality of the virome. Phage populations may be more complex than a mere reflection of bacterial communities, making it likely they contribute directly to disease development, including inflammatory bowel disease (IBD)^42,50^ and other GI conditions. Conversely, it may be possible to reinvent/repurpose centuries-old phage therapy to deliver health benefits by, for example, returning the patient to a eubiotic state, which is the intention of FMT. However, currently there is little consideration of viral populations in such approaches and it is imperative that donor screening of viromes as well as all components of the gut microbiota is implemented in this age of precision medicine.^51^

## Coevolutionary dynamics of gut bacteriophages and bacteria

**Figure 1.**
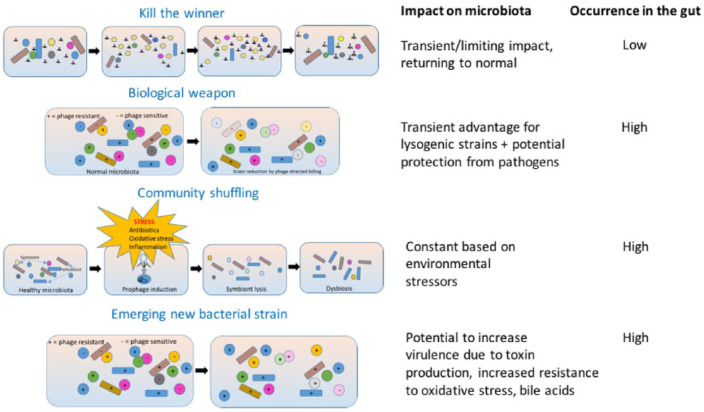
**Proposed mechanisms of phage-driven intestinal dysbiosis.** In the ‘Kill the Winner’ model, phages target and kill dominant commensal bacteria that are usually growing the fastest, thus reducing their numbers in the GI tract. In the ‘Biological Weapon’ model, commensal bacteria use the phages they carry as weapons to kill competing bacteria, causing a decrease in bacteria, leading to dysbiosis. The ‘Community Shuffling’ model proposes that environmental stressors such as antibiotic therapy, oxidative stress or inflammation can trigger the introduction of prophage into bacteria, resulting in lytic infection of symbiotic bacteria, altering the relationship between symbionts and pathobionts. The ‘Emerging new bacterial strain’ model suggests the potential to increase virulence through acquisition of genetic material – in effect establishing lysogeny in the host rather than inducing lysis.

Several hypothetical models have been proposed to account for phage-driven intestinal dysbiosis (Figure 1).^52^ In the ‘Kill the Winner’ model, phages target and kill dominant commensal bacteria that are usually growing the fastest, thus reducing their numbers in the GI tract. The model relies on the specific bacterial population being relatively high within the community as phages cannot actively move and so rely on opportunistic contact with their bacterial host (or prey) in order to infect and reproduce. This approach is therefore only a consideration for dominant members of an ecosystem, with only limited evidence of this occurring within the gut.^53^ Even contrived models of the gut microbiota, with the composition stacked towards a single bacterial strain (*E. coli*) alongside coliphage infection, fail to demonstrate the phenomena.^54^ It is possible that the physical structure and physiological nature of the gut environment restricts such interactions and protects bacterial populations from phage contact.

Another mechanism is the ‘Biological Weapon’ model, whereby commensal bacteria use the phages they carry as weapons to kill competing bacteria, leading to dysbiosis. By killing competitor bacteria, phages indirectly benefit their host. This model may play an important role in protection against pathogens, although additional experimental evidence is lacking. Within the gut environment, competition experiments between lysogen and sensitive strains of *E. faecalis* in monocolonized mice showed a transient enrichment of lysogen over sensitive strains.^55^

The ‘Community Shuffling’ model proposes that environmental stressors such as antibiotic therapy, oxidative stress or inflammation trigger the introduction of prophage into bacteria, resulting in lytic infection of symbiotic bacteria, altering the relationship between symbionts and pathobionts.^56^ Subinhibitory concentrations of certain antibiotics, including quinolones or beta-lactams, can drive this phenomenon in various gut bacteria.^57^ Inflammation, through the exacerbation of oxidative stress, may also be responsible for prophage induction.^50^

In addition to the three models described, which rely on the phages’ ability to lyse their hosts, phages can also transfer genes to bacteria to modify their phenotype, which is seen in the ‘Emerging New Bacterial Strain’ model – in effect establishing lysogeny in their host rather than lysing it. Within the complex environment of the gut, what drives these differential behaviours is unknown, but it is vital to understanding the phage–bacterial ecosystem. Several gut metagenomics studies have highlighted the potential of the virome to confer antibiotic resistance,^10,45^ and that this behaviour can facilitate the transfer of large bacterial DNA segments between strains. Further supporting this theory, it has been shown that the bacteriophage are able to control bacterial biological functions through the bacteriophage transcription factors during the lysogenic cycle. Specifically, it has been shown that during lysogeny the phage transcription factor Cro can activate the enterohaemorrhagic *E. coli* type III secretion system (T3SS), which has been shown to enhance the virulence of the bacteria.^58^ Mathematical modelling of the interaction suggests that the nested infection networks affecting phage and bacteria dynamics may involve a much more complex multi-type Lotka–Volterra framework.^59^ This logistic equation mathematically plots population growth when two species are competing for similar resources within the same environment.

Conversely, gut bacteria simultaneously evolve and develop microbial defence mechanisms against predatory bacteriophages. The most well studied is the restriction modification system involving bacterial restriction endonucleases which cleave double-stranded phage DNA.^60^ To prevent destruction of its own DNA, methyl groups are added.^61^ Bacteria can also block or hide their membrane receptors to limit phage propagation,^62^ or increase production of competitive inhibitors that render phage receptors unavailable to phage docking.^63^ As a failsafe, bacteria can also self-destruct by ‘abortive infection’, preventing spread of the infection to neighbouring cells.^64^

The bacterial defence mechanism currently gaining most interest in the context of the gut microbiota is CRISPR-Cas.^65,66^ CRISPR (clustered regularly interspersed palindromic repeats) loci together with their associated *cas* genes provide acquired immunity and defence by bacteria. CRISPR-Cas interfere with phage replication and can play a significant role in microbial community structure, including within the gut microbiota, although they are not infallible.^67^ The importance of this phenomena within the gut microbiota requires deep sequencing metagenomics studies to allow CRISPR-Cas systems to be studied.

These evolutionary survival tactics by the bacteriophages and bacteria makes for a dynamic and constant arms race, often referred to as the Red Queen race/hypothesis in which both parties are ‘continuously running to stay in the same place’ or evolving in equal measure but keeping pace with each other, resulting in a zero-sum game. In an experimental model that highlights this, a novel bacteriophage, p10 (related to Myovirus Felix O1), its host *E. coli* strain LF82 and an *E*. *coli* strain MG1655 (to which p10 cannot bind), a virulent bacteriophage was able to adapt by utilizing the gut microbiota to mutate and infect the *E*. *coli* strain MG1655 within a murine gut but not *in vitro*. This suggests that the gut microbiota are key intermediates for phage mutations, allowing them to switch host and to exist.^68^ Understanding more about the mechanisms that maintain relationships and the balance between bacteriophages and bacterial populations in the gut is crucial in understanding the perturbations that occur in the healthy gut microbiota which result in diseased states such as IBD and cancer.

Most studies have focused on the dysbiotic process in bacterial populations, wherein there is a shift from symbiotic to pathogenic. Four different scenarios can occur with respect to the bacteriophage and the bacterial population which can either suggest manifestations of disease or stable equilibrium states leading up to inflammation. If the bacterial richness increases with a parallel increase in the richness of the bacteriophage component, it would signify that the latter are proliferating simply as a result of more host bacteria to prey open. Conversely, a decrease in both components could be interpreted as scarcity of bacterial prey adversely impacting the bacteriophages. However, an increase in bacteriophage richness with a concomitant decrease in bacterial richness has a totally different connotation as it implies that the former is the driver and orchestrator of these changes. This pattern of changes has been documented in IBD in the seminal paper by Norman and colleagues.^42^ Finally, the combination of increased bacterial richness accompanied by a decrease in bacteriophage richness implies that the bacteria have a survival advantage and are the initiators of change. It is imperative that robust disease definitions are adhered to in disease conditions to elucidate the exact nature of the perturbation of the intricate relationship of bacteriophages and bacteria in the gut.

The recent increase in metagenomic studies will allow these interactions to be interrogated in more detail in the coming years. This approach has already led to discovery of hitherto unknown viruses. A particularly striking case of a virus discovered via metagenomics is crAssphage, which is by far the most abundant human-associated virus known, comprising up to 90% of sequences in the gut virome.^69^ The significance of these viruses in health or disease is still not clear.

## The gut virome in GI diseases

**Figure 2.**
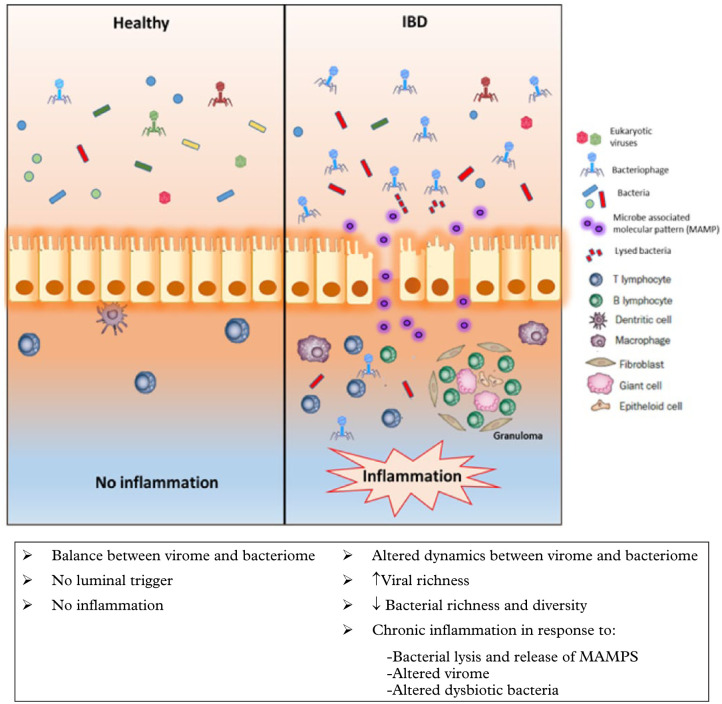
**Schematic representation of the alteration of the enteric virome and bacteriome in inflammatory bowel disease.** There is an expansion of bacteriophages with increased richness of the gut virome and an associated decrease in richness and diversity of the gut bacteria leading to ‘microbial dysbiosis’, which could be the trigger for chronic inflammation in IBD. Alteration of the viral–bacterial dynamics may also lead to increased bacterial lysis and release of microbe-associated molecular patterns (MAMPs) that could attract inflammatory cells in the lamina propria. The luminal changes could also be an ‘epiphenomenon’ as a result of the inflammatory cascade.

When considering the role of the enteric virome in the aetiopathogenesis of GI diseases, the bridge from association to causation needs to be crossed. Enteric eukaryotic viruses and bacteriophages can kill host cells and bacteria respectively to release potential activators of chronic inflammation. The role of the bacterial microbiome as a possible intermediary in this interaction has been documented in *Atg16/1*-deficient mice, where murine norovirus induced pathological changes that were akin to Crohn’s disease (CD), a chronic relapsing and remitting IBD, and these changes were abolished with antibiotics.^70^ A further experimental model has shown that pretreatment with an antiviral cocktail caused mice to display more severe dextran sulphate sodium-induced colitis when compared with mice that were not pretreated, highlighting that the virome may play a protective role against colitis. Furthermore, they found mice that were reconstituted with TLR3+7 agonists had resolution of their colitis and suggested that the resident intestinal virome through TLR3- and TLR7-mediated IFN-β secretion by plasmacytoid dentritic cells plays a protective role in gut inflammation.^71^ These experimental model suggests the importance of the virus–bacterial-host interactions in GI diseases and highlights the importance of viruses as initiators of inflammation (Figure 2). However, evidence for specific viruses acting as triggers for IBD has not been validated in human studies^72^ and a full understanding of the role of the virome is needed.^73^ With newer metagenomic methods it is now possible to study the ‘entire virome’ as a cohesive ecological unit that can change as a whole and impact on host immunity and result in disease. The entire viral genetic material can now be assessed, but a large proportion of this cannot be correctly annotated as the viral database is still quite rudimentary. With increasing additions of sequences in the coming years, the identification and understanding of the gut virome will continue to improve.

### Inflammatory bowel disease

Evidence suggests that a core healthy gut phage community is reduced or altered in patients with IBD (Table 2).^74–76^ This was first suggested by Lepage and colleagues, who identified higher numbers of bacteriophages by epifluorescence microscopy in mucosal samples of patients with CD compared to controls.^50^ Transmission electron microscopy (TEM) identified bacteriophages as members of the Siphoviridae, Myoviridae and Podoviridae, which was confirmed by DNA sequencing. It was noted that patients with CD had more VLPs per biopsy than healthy individuals. Additionally, there were fewer VLPs per biopsy from ulcerated mucosa of CD patients than non-ulcerated specimens. In an experimental model it has been shown that the gut inflammation is the driver for bacteriophage transfer; specifically, it was shown that transfer of the prophage SopEΦ between two *Salmonella* Typhimurium strains SL1344 and ATCC14028S, was increased in the presence of gut inflammation, with a >55% lysogenic conversion to ATCC14028S compared to a lysogenic conversion reduction of 10^5^ in the absence of any inflammation.^77^

**Table 2.** Summary of studies assessing the enteric virome in IBD patients.

Study	Year	Patient cohort	Age group	Sample source	Number of patients	Number of controls	Method	Key findings
Lepage et al.	2008	CD	Adult	Colonic biopsies	19	14	Epifluorescence microscopy, transmission electron microscopy	↑Bacteriophages detected in the mucosa from CD patients than from healthy individuals
Wagner et al.	2013	CD	Paediatric	Ileal and colonic biopsies, gut wash samples	6, 3	8	Viral metagenome – 454 pyrosequencing Roche GS-FLX Titanium	Differences in bacteriophage composition between CD patients and control individuals
Pérez-Brocal et al.	2013	CD	Adult	Faeces, ileum tissue	11, 1	6	Viral metagenome – 454 pyrosequencing Roche GS- FLX titanium plus	↓ Diversity of viral and bacterial communities in CD samples compared with the control group ↑ Variability between the CD samples in both virome and microbiome
Wang et al.	2015	CD + UC	Adult	Colonic tissue (biopsy/surgery)	10	5	Viral metagenome – Illumina HiSeq 2000 sequencing platform	↑ Viral sequences in CD Difference in abundance and diversity within the virome between CD and control group
Norman et al.	2015	CD + UC	Adult	Faeces	UK Cohort (UC 21, CD 14) Chicago cohort (UC 17, CD 8) LA cohort (UC 22, CD 1) Boston cohort (UC 15, CD 20)	UK Cohort (HC 22) Chicago cohort (HC 24) LA cohort (HC 0) Boston cohort (HC 10)	Viral metagenome – Roche 454 (initial study) and Illumina MiSeq platform (in-depth analysis)	↑ Viral richness and Caudovirales expansion in CD and UC ↓ Decreased bacterial richness and diversity in UC and CD Inverse correlation of Caudovirales with prevalent bacterial taxa in CD

UC, ulcerative colitis; CD, Crohn’s disease; HC, Healthy controls.

Metagenomic surveys have confirmed these virome alterations in patients with CD.^74,78,79^ Ileal biopsies, colonic biopsies and gut washes from paediatric CD patients and ileal biopsies from control patients^78^ identified the same three Caudovirales family members identified by TEM.^50^ Although the largest number of virus sequence matches were found in the patients with CD, the study lacked an in-depth analysis of differences in richness and diversity of the gut virome in these patients. This finding was replicated by Wang and colleagues in their analysis of colonic biopsies from IBD patients and controls, wherein a difference in both abundance of viruses and diversity within the virome was noted.^79^ There was a suggestion that viral abundance and diversity were associated with differences in the bacterial composition within the colon, suggesting a role of the gut virome in bacterial dysbiosis.

The report from Pérez-Brocal, on the other hand, was the first to use metagenomics to systematically analyse the correlation of the viral and bacterial components of the microbiome in patients with CD.^74^ The study confirmed the presence of the same three dominant Caudovirales family members but in contrast showed decreased diversity and abundance of viral sequences in patients with CD as opposed to controls. They also found a parallel decrease in diversity and abundance of bacteria in CD patients, which has been replicated in multiple other studies. One of the key findings from the analysis of viral sequences was an over-representation of *Synechococcus* phage S CBS1 and Retroviridae family of viruses in CD patients, identifying them as potential disease biomarkers.

A more definitive study highlighting the interaction of the gut virome and its intrinsic interplay with bacteria was carried out on a cohort of UK IBD patients and their healthy household contacts [12 household controls, 18 CD and 42 ulcerative colitis (UC)] and subsequently validated in two US IBD cohorts.^42^ A significant expansion of Caudovirales bacteriophages in both CD and UC patients was noted. No such distinction was noted with richness or diversity of Microviridae, suggesting that the bacteriophage increase was restricted to certain taxa. Significant reductions in bacterial diversity and richness were observed in both CD and UC patients, which was inversely correlated with the expansion of Caudovirales bacteriophages. This led to speculation that the viral changes were the primary driver in the process, with secondary shifts in the bacterial population. The exact reason for the expansion of the Caudovirales bacteriophages was unknown, but could have been related to diet or activation of prophages from commensal gut bacteria. Although Caudovirales bacteriophages expanded in both CD and UC patients in the US cohorts, the specific relationships between bacteriophage and bacterial taxa seen in the UK cohort were not seen. Bacteroidaceae bacterial families were correlated inversely with several Caudovirales taxa in CD, but Caudovirales were positively correlated with Enterobacteriaceae, Pasteurelloacaeae and Prevotellaceae in CD. These correlations were not present in UC patients, suggesting a (environmental/lifestyle) distinction between these two subgroups of patients.

The relationship of phages and their bacterial ‘prey’ was studied in IBD patients from the cohort of Norman and colleagues, with specific reference to *Faecalibacterium prausnitzii*.^80^ A higher occurrence and proportion of some *F. prausnitzii* temperate phages in patients with IBD suggests that this increased activity is possibly related to the depletion of *F. prausnitzii.* This result is clinically very pertinent since IBD patients have been documented to have a lower abundance of *F. prausnitzii* in their microbiota.^81^ The mechanistic relationship of the phages and their bacterial hosts needs to be studied to elucidate the causative role of the virome in IBD.

Most studies on gut viral populations have focused on DNA viruses, of which bacteriophages make up the largest cohort and the methods utilized do not address or include RNA and enveloped viruses.^42^ This obvious deficiency needs to be addressed as many pathogenic enteric viruses are RNA viruses. However, a small study on two normal participants found that the majority of RNA viruses detected in the faeces were plant pathogens and most likely diet-derived.^82^ The likelihood of such dietary-acquired bacteriophages acting as triggers for changing gut bacterial populations and initiating inflammation is an attractive hypothesis that needs to be validated. Conversely, the transient contact of diet-derived RNA with the bacterial population in the lumen and the gut immune system brings into question their importance in the pathogenesis of IBD.

## Current microbial therapeutics and consideration of the virome

With recent advancements in sequencing technology the diversity of the enteric human virome is being increasingly revealed, leading to new possibilities for altering the gut microbiota to prevent or treat disease or to reduce disease risk. Recognizing that the gut microbiota can be manipulated by diet, smoking, prebiotics, antibiotics, probiotics, synbiotics and FMT,^83^ attempts have been made to restore human health utilizing a variety of these approaches. However, there is a paucity of data reflecting their impact on the gut virome.

FMT is a successful treatment for refractory *Clostridium difficile* infection (CDI), with cure rates of 87%.^84^ FMT also shows promise in IBD, although additional studies are required as current findings indicate donor selection is crucial for successful outcome.^85–88^ While shifts in bacterial communities using FMT are well established, less is known on its role in altering the gut virome and its influence on disease activity. A recent study assessing FMT in the treatment of CDI showed that CDI patients who responded to FMT took on a more significant donor-derived enteric virome component compared to nonresponders. In addition, all recipients infused with donor faeces containing greater Caudovirales richness than the recipient were cured.^51^ Furthermore, it suggested that responders to FMT has significantly lower eukaryotic virome richness than nonresponders at baseline.^89^ These studies highlight that restoration of virome communities are as important as the bacterial component, with donor selection – based on virome characteristics – needing to become more considered. A further study reported outcomes following treatment of CDI using sterile faecal filtrate transfer.^90^ This filtrate contained only the bacterial debris, proteins, antimicrobial compounds, metabolic products and oligonucleotides/DNA rather than intact microorganisms. Following the faecal filtrate administration, faecal samples were dominated by *Lactococcus* bacteriophages and the phageome of the patient was substantially altered, which persisted after 6 weeks. Broecker and colleagues used FMT to treat CDI and found phages were equally abundant in the treatment-responsive patient and donor.^91^ They also found that a healthy microbiota appears to be characterized by low phage abundance and that patients receiving FMT established a virome that was similar to that of the donor.^47^ They therefore suggested that FMT transferred a core population of viruses. Significantly, on longer-term follow up they found phage communities of the treated patient remained similar to those of the donor in composition diversity and richness. These studies suggest that FMT may in some way alter the gut virome, but limitations in our ability to fully characterize the gut virome limits our understanding of how they are transferred and their specific role in response to FMT.

The metagenomic assessment of the gut virome has also been performed in a solitary patient with refractory CDI who was followed up over a period of nearly 5 years after FMT.^47,92^ In this study, the virome profile remained stable and donor-like over the period of sequential follow up, suggesting stabilization of the gut virome over time. There are no definitive phages identified against *C. difficile* so far and this may preclude their definitive role in causation of this infection.^93^ However, the virome may play a role in clearance of CDI, as the study by Zuo and colleagues showed that recipients treated with donor faeces consisting of a greater richness of Caudovirales than that of the recipient were all cured with FMT.^51^

Only a single study to date has reported on changes in the virome of FMT in the context of IBD. The study by Chehoud and colleagues investigated the impact of a single healthy adult donor and three paediatric UC recipient patients who received 22–30 FMT treatments over the course of 6–12 weeks. Using metagenomics, samples from the donor and the three recipients (before, during and after FMT treatment) were assessed and showed that treatment of UC using FMT was associated with the transfer of numerous temperate phages, but no viruses corresponding to pathogenic viruses that infect human cells were detected.^94^ The authors proposed that the transfer of phages was a characteristic of FMT although the stability of phage populations or the long-term clinical significance of phage transfer requires further investigation.

## Conclusions/perspective

Characterization of the gut virome is still in its infancy, with a requirement for new viruses to be identified and characterized in order that its full functional impact can be defined. We are a very long way from proving Koch’s postulates of disease causality for the gut virome and its constituents. Major questions remain in order to allow the field to move forward (Box 2). The continued evolution of high-throughput sequencing accessibility is allowing our understanding of the gut virome to increase exponentially, but this still lags behind our ability to interrogate other components of the gut microbiota. The ‘elephant in the room’ remains that our understanding of viromes to date is principally built and based on fragments of sequences and fragmented genomes of prophages which may not generate productive/infective phages. It is highly possible that the gut virome may be an undiscovered entity in understanding disease pathways that contribute to the inflammatory process. However, in order to discover this, consideration of the gut virome needs to parallel that of gut bacterial and fungal diversity. Once the intrinsic tripartite relationship between the three components of the gut microbiome is clearly understood and its alteration documented in IBD, the field of microbial-based IBD therapeutics will be transformed.

**Box 2.** Major questions that remain to be answered in order to allow the gut virome field to move forward.

**1. How should controls be selected?** What is an appropriate control/comparator group for virome studies in different healthy or diseased cohorts? Such a group needs to account or exclude key confounders of individual microbiome variability, such as age, sex, lifestyle, environment, geography and behaviour that can all to some extent impact on the structure and function of microbiomes in the short or long term. The use of related individuals living in the same household and environment may be a better comparator group than unrelated randomly sourced individuals. **2. What sampling approach to use?** Cross-study comparisons of datasets is difficult if not impossible due to differences in sample collection, handling, storage and processing (e.g. VLP isolation, DNA/RNA extraction, sequencing platforms and parameters). There is an urgent need to develop standardized protocols and establish Gold Standard procedures and protocols.^95^ **3. Viromics.** There is currently no open-access, easy-to-use bioinformatics pipeline that uses raw sequence reads that can remove host DNA, search for bacterial contaminants, and assign taxonomy and functionality to viruses within a sample. Innovative viromics tools have recently been described for characterizing aquatic viromes (e.g. iVirus^96^), although their application to human virome analyses is limited by the need to incorporate modifications to filter out nonviral sequences.
